# Patient and health professional views on rehabilitation practices and outcomes following total hip and knee arthroplasty for osteoarthritis:a focus group study

**DOI:** 10.1186/1472-6963-10-119

**Published:** 2010-05-11

**Authors:** Marie D Westby, Catherine L Backman

**Affiliations:** 1 Rehabilitation Sciences Research Graduate Program, Faculty of Medicine, University of British Columbia, Vancouver, Canada; 2 Mary Pack Arthritis Program, Vancouver Coastal Health, Vancouver, Canada; 3 Department of Occupational Science and Occupational Therapy, Faculty of Medicine, University of British Columbia, Vancouver, Canada; 4 Arthritis Research Centre of Canada, Vancouver, Canada

## Abstract

**Background:**

There is worldwide variation in rehabilitation practices after total hip arthroplasty (THA) and total knee arthroplasty (TKA) and no agreement on which interventions will lead to optimal short and long term patient outcomes. As a first step in the development of clinical practice guidelines for post-acute rehabilitation after THA and TKA, we explored experiences and attitudes about rehabilitation practices and outcomes in groups of individuals identified as key stakeholders.

**Methods:**

Separate focus groups and interviews were conducted with patients (THA or TKA within past year) and three health professional groups: allied health professionals (AHPs), orthopaedic surgeons, and other physicians, in Canada and the United States. Pairs of moderators led the focus groups using a standardized discussion guide. Discussions were audiotaped and transcribed verbatim. A content analysis within and across groups identified key themes.

**Results:**

Eleven focus groups and eight interviews took place in six sites. Patients (n = 32) varied in age, stage of recovery, and surgical and rehabilitation experiences. Health professionals (n = 44) represented a range of disciplines, practice settings and years of experience. Six key themes emerged: 1) Let's talk (issues related to patient-health professional and inter-professional communication); 2) Expecting the unexpected (observations about unanticipated recovery experiences); 3) It's attitude that counts (the importance of the patient's positive attitude and participation in recovery); 4) It takes all kinds of support (along the continuum of care); 5) Barriers to recovery (at patient, provider and system levels), and 6) Back to normal (reflecting diversity of expected outcomes). Patients offered different, but overlapping views compared to health professionals regarding rehabilitation practices and outcomes following THA and TKA.

**Conclusion:**

Results will inform subsequent phases of guideline development and ensure stakeholders' perspectives shape the priorities, content and scope of the guidelines.

## Background

Total hip arthroplasty (THA) and total knee arthroplasty (TKA) surgeries are highly successful orthopaedic procedures for more than 62,000 Canadians [1] and 773,000 Americans [2] each year. The growth in number of THAs and TKAs exceeds the aging of our population due in part to both younger and older individuals electing joint replacement surgery as a feasible option for their advanced hip and knee osteoarthritis (OA) [3].

Nearly all patients receive post-operative physical therapy and/or other rehabilitative services in the hospital, as an outpatient or through home care services [4]. However, the setting, timing, amount and treatment approaches differ widely [5-8]. Despite the cost effectiveness of THA and TKA, in-hospital and rehabilitation costs associated with these surgeries place significant burdens on North American healthcare systems [2,9-11]. Rehabilitation interventions (e.g., physical therapy, occupational therapy, nursing care) may enhance surgical outcomes; however, their precise contribution to long-term outcomes such as physical function, mobility, participation in life roles and health-related quality of life (HRQoL) is not clear. A National Institutes of Health (NIH) conference concluded that "...rehabilitation services are perhaps the most understudied aspect of the peri-operative management of TKA patients" [12].

Disparate views on need for total joint arthroplasty (TJA) surgery, expectations and outcomes of surgery have been reported for physicians and patients [13-15], and between surgeons and other health professionals [16]. Hewlett suggests that patients' assessments may differ from those of health professionals due to the influence of needs, attitudes, priorities, experiences and expectations [17]. It is therefore necessary to explore patient and provider expectations to inform clinical practice guidelines.

The Canadian health care system is characterized by universal access and government funded health care for physician and hospital-based services, few for-profit providers, and lower national health care expenditures than in the US [18], with its varied access to public and private providers depending on one's insurance. These differences in turn influence surgical wait times [1], access to and funding for rehabilitation services, and health outcomes [18]; thus the need to incorporate both perspectives.

The purpose of this study was to move beyond the existing literature and explore patient and health professional experiences with current rehabilitation practices and outcomes following THA and TKA to inform the development of clinical practice guidelines applicable for North America.

## Methods

### Sampling frame

We were interested in perspectives from four stakeholder groups: 1) individuals who had a primary THA or TKA for OA within the past year; 2) allied health professionals (AHPs, e.g., physical therapist (PT), occupational therapist (OT), nurse, medical social worker) currently providing THA or TKA rehabilitative care, education or counseling; 3) physicians (e.g., rheumatologist, physiatrist, family practitioner) who provide THA or TKA care; or 4) orthopaedic surgeons currently performing THA or TKA. Patients were excluded if they were less than 19 years of age, could not converse in English; or had undergone THA or TKA surgery for inflammatory arthritis, acute fracture/trauma or tumour. Spouses were permitted to join the patient discussion groups.

### Recruitment

We therefore used strategies to accrue a purposive sample across stakeholder group, demographics and level of experience. Notices, inviting interested individuals to contact the local study coordinator, were posted in clinics, waiting rooms, seniors' centers and arthritis consumer groups' newsletters as applicable to each stakeholder group. E-mail notices were distributed using staff directories for all types of health professionals.

### Focus Groups/Interviews

Focus groups are particularly suited to studying diverse perspectives to gain insight into participants' experiences [19,20] and were the primary means of gathering data, where possible. Focus groups encourage contributions from less verbal individuals who feel supported by other group members with shared experiences [21]. However, individual interviews were conducted when participants were unable to attend their group. Both focus groups and interviews have been used previously in studying various aspects of THA and TKA care, patient experiences and expectations [22-27], but we are not aware of studies that examine THA and TKA rehabilitation practices and outcomes from multiple stakeholders' perspectives.

A discussion guide was developed with input from a multi-disciplinary group of clinicians experienced in THA and TKA rehabilitation and researchers experienced in focus group methodology. Open-ended questions progressed from general and uncued to more specific questions with accompanying probes [20,28]. The discussion guide was tested twice and revised to improve clarity based on health professional and patient feedback. Key questions and probes [Appendix A] were rephrased for each stakeholder group to ensure relevance to participants [20]. Separate focus groups were conducted with each set of stakeholders to avoid a perceived hierarchy among mixed professional and professional-patient participants [29].

A pair of moderators led each focus group using the standardized discussion guide. The four moderators were female PTs with experience in TJA rehabilitation and group process and included the lead author. Prior to the first focus group, moderators were given written and videotaped instructions on focus group methodology, moderating tips and use of the data collection forms, and each pair conducted a pilot session to gain skill and confidence in moderating sessions and trouble shoot problems related to audiotaping, timing and logistics.

Focus group sessions lasted 90 minutes for health professionals and 120 minutes for patient groups (allowing for a stretch break). Individual semi-structured interviews (face-to-face or telephone) of 30-60 minutes were conducted with participants unable to participate in a focus group; they followed the discussion guide. Sessions were audiotaped and transcribed verbatim for analysis. Participants recorded thoughts on a response form prior to sharing their perspectives with other group members. Forms were collected and together with the moderators' field notes served to enrich transcripts and study rigor [30]. Member checking was incorporated into focus groups and interviews by inviting participant feedback on the moderator's summary of the session [21]. Immediately following each focus group, the moderators met to debrief, identify issues that may influence analysis and suggest possible modifications to the discussion guide [21].

Ethical approval was received from the UBC Behavioral Research Ethics Board and the Vancouver Coastal Health Research Institute for the primary site and as required by institutional policy for each of the other sites. All participants provided informed consent prior to participation, and were offered a small token ($10 gift certificate).

### Data analysis

**Figure 1:**
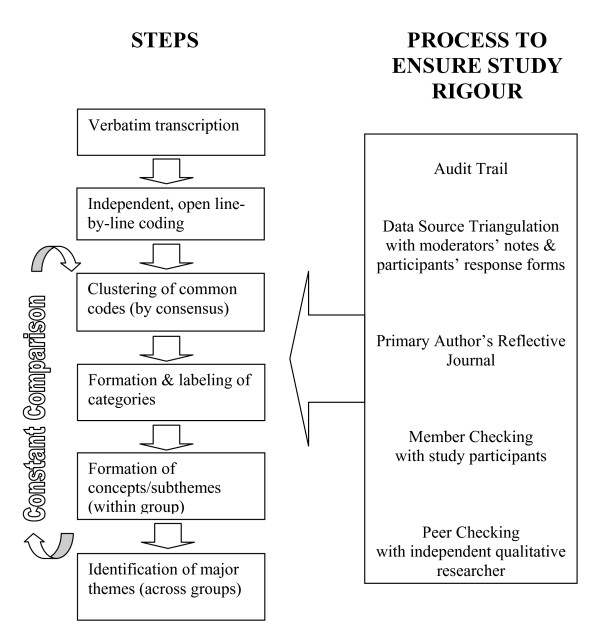
**Data analysis flow chart**.

A thematic content analysis occured concurrently with data collection to allow for revision of questions and development of new lines of inquiry [20,21,29,31]. After checking transcripts for accuracy, the two authors independently read the transcripts and performed line-by-line, open coding [29], and, following the process outlined in Figure 1, developed sub-themes for 'within group analysis' and subsequently refined these into key themes for 'across group analysis'. Disagreements in coding and categorization were discussed and the coding framework refined as necessary using a constant comparison approach [29]. Minority opinions or outliers (negative cases) were identified and discussed [30].

Data collection was discontinued when it was agreed that no new ideas or issues were likely to be raised [20,29]. A decision audit trail was maintained throughout the data collection and analysis phases. Once key themes were identified, transcripts were reviewed and representative quotes selected for each theme. Portions of the coding framework and final analysis were shared with an independent, experienced qualitative researcher for peer checking [30].

## Results

Eleven focus groups and eight semi-structured interviews were conducted in five Canadian and one US site. Participants included 32 patients and four spouses, 30 AHPs, five physicians and nine surgeons [Tables 1 &2]. Despite efforts to recruit an ethnically diverse sample, patients were primarily Caucasian but included one African American and one Aboriginal person living on reserve. Allied health professional groups included PTs, OTs, nurses, physician assistants, social workers, and a rehabilitation assistant and fitness professional. Physicians included family practitioners, physiatrists and a rheumatologist. Focus groups ranged in size from four to 10 participants.

**Table 1:** Patient participant demographics (n = 32)*

Patients (Type of surgery)	Age (Range, years)	Gender (♀/♂)	English as first language	Education (Some college or higher)	Post-op stage (Range, months)	Rehab status (Completed rehab)	Work status (Retired)	Lives in urban community
THA n = 13	46 - 81	7/6	13	10	1 - 11	7	8	9

TKA n = 19	46 - 78	11/8	18	15	1 - 10	9	10	14

* - patient participants only (does not include the 4 spouses)

**Table 2:** Health professional participant demographics (n = 44)

Professions	Age(Range, years)	Gender (♀/♂)	English as first language	**TJA experience**^**1**^**(Range, years)**	**TJA patient volume**^**2**^**(Cases/year)**	**Practice setting**^**3**^	Urban-based practice
AHPs n = 30	28 - 62	26/4	25	1 - 35	<50/yr = 7 50-100/yr = 8 >100/yr = 15	Inpt acute = 4 Inpt rehab = 4 Outpatients = 15 Home care = 5 Other = 2	22

Surgeons n = 9	33 - 64	0/9	7	1 - 30	50-100/yr = 1 >100/yr = 8	Teaching hospital = 8 Regional hospital = 1	9

Physicians n = 5	41 - 60	1/4	4	6 - 35	<50/yr = 1 50-100/yr = 2 >100/yr = 2	Inpt acute = 1 Inpt rehab = 1 Private practice = 3	5

AHPs = allied health professionals; TJA = total joint arthroplasty; Inpt = inpatient

1 - Years of experience providing surgical, treatment or counseling services to patients with THA or TKA

2 - Number of combined THA and TKA patients treated or operated on each year

3 - Number of professionals practicing in each setting; for AHPs "Other" = recreational setting

### Key Themes

Within group analyses for each stakeholder group resulted in the subthemes summarized in Tables 3, 4, 5 and 6. Further comparison using constant comparison across groups uncovered six major themes. Thus, subtheme labels reflect concepts specific to each group whereas the key themes reflect concepts across all participants. Quotes are attributed to participants by noting their age, gender and group, e.g., 41, F, FP is a 41 year old, female, family practitioner.

**Table 3:** Patient subthemes and sample quotes*

Subtheme 1: I wasn't expecting that.
"I didn't realize the enormity of the procedure or the aftermath, I really didn't. So it was kind of hard on me because I didn't realize the pain I was going to have." [78 F, THA]
"I asked everybody in physio. They slept an hour approximately a night for about 5 weeks. That was all. Like when you're in hospital you were doped up. As soon as you went home it was about an hour, and not just at a time. It was an hour in 24..." [46 F, TKA]
"Has anybody else had a little bit of depression after the surgery? Am I the only one? I would cry over anything." [76 F, TKA]
"It's exhausting for the spouse when this is going on. We've had a lot of tears and stress." [spouse of 65, M, TKA]
Subtheme 2: It takes all kinds of support
"Physics that explain and explain are so invaluable because we're all going through such anxiety." [64 F, TKA]
"My primary care doctor was a great support. The surgeon was motivating, believes in you..." [61 F, TKA]
"...if I had this to go through again I would have somebody at home, because I certainly could have used a little help at home." [76 F, THA]
"...the other patients. I mean I'm basically modeling myself on all their efforts too. I think it served to sort of propel me to become more motivated." [52 M, TKA]

Subtheme 3: My body, my responsibility
"I think you really have to have a kind of a very positive attitude. It's not easy. It's not easy when you have a lot of pain." [75 M, TKA]
"You really have to advocate strongly for yourself and the services. It's not something that is openly offered. It's a matter that you have to pursue [52 M, TKA]
"If the surgeon does his part, I should do mine. I did exercises 8 to 9 months before surgery so felt confident going for surgery. I quit smoking and walked regularly [57 F, TKA]

Subtheme 4: Back to normal
"...to not be able to do that job anymore would be the saddest thing in my life." [46 F, THA]
"The biggest thing for me is getting my walking ability back to where it was say five years ago." [73 M, TKA]
"Getting up and running, jogging, whatever, riding a bike. I didn't really talk to [my surgeon] about it. I really wanted to discuss playing ice hockey but that is totally out of the question, and that was my goal..." 51 M, TKA]
"Being so happy that your personality has returned. Because I'm sure that we've all had varying degrees of changes over the years just in learning how to live and manage the pain. You can walk around with a smile on your face and probably all of us feel 10 to 20 years younger." [46 F, THA]

* Findings based on 5 focus groups and 2 interviews. Legend: Panelists are identified by age, gender (M = male, F = female) and type of surgery (THA or TKA)

**Table 4:** AHP subthemes and sample quotes*

Subtheme 1: We all need to be on the same page
"It's so hard to get information about the type of surgery... it's like pulling teeth. So lack of information is problematic and it's one of the frustrations I think most therapists face." [41 F, PT]
"...because we're small, we can call up one person... so it's easy. I think it works well, the link from the communication we have, acute hospital stay to community back into the outpatient department." [44 F, OT]
Subtheme 2: We each have a role to play
"The patients themselves - just their attitude, their motivation. We see people for pre-op and I think 'Oh, it's going to be terrible when they have their surgery and they come back.' Right away I can tell this person's going to have a hard time." [39 M, PT]
"...there's a contract between the patient and myself. They've actually given something up and I have taken it from them, so there's a bit of an obligation there as a professional to make sure I give back to them the value for what they're paying for." [61 M, PT]

Subtheme 3: Patients need lots of support
"...people motivate each other. They can compare notes, etcetera, but sometimes the comparison can work negatively in that they'll say 'Oh well, I had a hip surgery by Dr. whoever and I'm at this week and I'm no where near where you are'." [41 F, PT]
"...it's really inconsistent among physicians in terms of who gets referred to home care and who gets referred to outpatient. There's no consistency... especially between health regions." [31 F, OT]

Subtheme 4: Barriers to patients reaching their full potential
"...some patients run out of physical therapy appointments. You know, their insurance only pays for 12 a year or something, and so you hit the 12 mark and there's not a whole lot you can do except for rely on them to do the exercises at home, and it can be a major impediment." [30 F, PA]
"...the [public] system the way that it's designed doesn't really follow through long enough. I'd like to have a six-month follow-up with these patients because I believe that most of the improvement that they see will occur in that early time. There are some gaps and I believe people sometimes don't reach their potential because of those gaps." [61 M, PT]

Subtheme 5: Rehabilitation is a continuum
"We get a lot of feedback from patients that tell us that getting to see the physical therapist [pre-op] and sort of train for the experience as though it's a sporting event and they have to be in shape for it... so that they're in shape to cope with what happens after the surgery." [53 F, RN]
"...phases I and II in the hospital where we have our rehab and then they go into the maintenance phase, which usually is within the community. Maybe we need to work together more as a whole, from surgery on and having those different phases available to the clients." [28 F, KIN]

Subtheme 6: Being able to do whatever you want to do
"Decreased pain, because I think that's the thing that people most want to get rid of. Whether that's with activity or just in standing, it isn't really relevant as long as whatever they're doing is pain free." [40 F, PT]
"It would be really nice if they came through the whole process without looking upon it as an enormous nightmare, that things have gone smoothly. You can't always predict everything I realize, but that they had a sense of confidence and a sense of control in the whole thing so that it's been a worthwhile process." [44 F, OT]

*Findings based on 4 focus groups and 1 interview. Legend: Panelists are identified by age, gender (M = male, F = female) & profession (PT = physical therapist, OT = occupational therapist, RN = nurse, SW = social worker, PA = physician assistant, KIN = kinesiologist)

**Table 5:** Surgeon subthemes and sample quotes*

Subtheme 1: Communication is the key
"...there's often times not enough communication between the orthopaedic surgeon and the therapist, the internist, the physical medicine doc, so that poses a particular difficulty" [36 M, SURG]
"A good part of healing is communication between provider and the recipient." [56 M, SURG]
Subtheme 2: Different expectations
"I think that as I'm learning as I'm going through, the expectations of a patient and the expectations of the physician are often different. They may not be well communicated at all times." [33 M, SURG]
"...depending on how much time you have to spend with people and so on. You may miss the boat in terms of what they're expecting." [>55 M, SURG]

Subtheme 3: Professsional support
"...what I do in my practice is tell patients that when I put a total joint in you, follow up is extremely important. It is the duty of the surgeon to maintain contact with his patients." [64 M, SURG]
"...by three months, I can usually determine how people are going to do and either reassure them and send them off or follow up on an as needed basis only... I think it's probably a waste of time to bring people who were doing well at discharge back for a long term follow-up." [56 M, SURG]

Subtheme 4: Barriers to recovery
"So you play this game with the insurance company and you get caught in the middle of the game as a patient...One of the biggest changes we've seen is with rehab. You know, only a certain patient population can now go to rehab and it's not the population you'd think." [33 M, SURG]
"The other thing that's non-existent for the most part is home physical therapy for the debilitated patient or the patient who is unable to get transportation somewhere or has social issues that would preclude them from being able to get to therapy. Those patients fall through the cracks, and for them it's a huge issue." [36 M, SURG]
"...approaches from physiotherapists vary greatly so that I don't refer anybody to a therapist for any purpose without knowing what their approach is." [56 M, SURG]

Sub-theme 5: Outcomes
"Well, the main indication for joint replacements is disabling pain and stiffness, and so the most important outcome is pain relief." [56 M, SURG]
"I recognize that it's a professional conflict to a certain extent but the ultimate responsibility for the outcome falls to the hands of the surgeon and if the therapist from the patients' perspective makes them worse or doesn't do a good job, it doesn't really matter because they still blame the surgeon in a sense for their poor outcome." [55 M, SURG]

* Findings based on 2 focus groups and 2 interviews. Legend: Panelists are identified by age, gender (M = male, F = female) and profession (SURG = surgeon)

**Table 6:** Physician subthemes and sample quotes*

Subtheme 1: Pain management
"I think that GPs think that [patients] shouldn't have pain. Or that the pain is trivial, unless they've had a knee replacement themselves when they know different." [60 M, RHEUM]
"Patients shouldn't be worried about becoming addicted to narcotics. It's a very rare individual that this is truly a problem for." [62 M, FP]
Subtheme 2: Continuity, coordination and communication
"...the very nature is that's what we do in the US is we don't really communicate well, is the lack of consistency and the absence of protocols or consensus. Sadly, you know, the paradigm of health care in the US is that there's such a vacuum." [47 M, PHYS]
"It's a bit of a grey zone and there's a fair amount of variability. So I usually try to gather information from [the patient's] surgeon through the patient and then from their physio...: [41> F, FP]

Subtheme 3: Access to rehab services
"...as you move out away, things become less and less available, and that applies to both community care as well as outpatient programs. And certainly if you're more in the hinterland access becomes a greater issue." [63 M, PHYS]
"...transportation is a big limiting factor." [47 M, PHYS]
"...it's particularly an issue for seniors that are on limited income. They will try to limit their physical therapy appointments because of finances. So they might not be getting quite as good of a result beyond their surgery." [41 F, FP]

Subtheme 4: Different patients, different needs along the continuum
"It used to be that people would cope with an awful lot and go soldiering on and feel that this is just the way it was. I'm seeing younger people now who come in and say, 'No, I'm not prepared to do this anymore. You know, I want to be able to do X and Y and so on, and I think I need to have something done.'" [60 M, RHEUM]
"There's considerably less need for rehabilitation in our experience with hips and considerably more for the knees. ... we just find that there tend to be more pain issues and more balance and control issues after total knee than after total hip procedures." [63 M, PHYS]

Subtheme 5: Outcomes
"Balance is a very important issue that needs to be followed... because safety issues and certainly preventing falls is going to be something that's very important to patients as well as the health care system itself." [63 M, PHYS]
"It's to get back to work, and then to get back to their activities that they like to do - so golf, swimming. You know, their premorbid activities that they like." [41 F, FP]

* Findings based on 1 focus group and 4 interviews. Legend: Panelists are identified by age, gender (M = male, F = female) and profession (FP = family practitioner, PHYS = physiatrist, RHEUM = rheumatologist

### Theme 1: Let's talk

A substantial amount of focus group time was spent discussing communication issues. The greatest energy and strongest group interaction occurred over the issues of inter-professional communication and collaboration across settings and throughout the continuum of care. While participants offered descriptions of both positive and negative patient-provider and inter-provider communication, most examples described how poor or lack of communication decreased efficiency, effectiveness and collaboration.

*"Communication amongst all the people involved is pretty much non-existent. There's no communication between surgeons and family doctors anymore."* [41 F, FP]

*"So we have this parade of people with total hips, for example, coming through as though they're all the same and they're not. And I think there's a real need for us all to get better information from the surgeon and I've crowed about this for a long time and it hasn't yet happened, but I think that's a major weakness.... I think this lack of information leads to rote [physical therapy] procedures that don't have very much thinking going on with them." *[62 M, PT]

Poor communication across settings (e.g., from in-patient rehab to family practitioner or private PT) was believed to contribute to inconsistent and poorly coordinated services and negatively impact clinical outcomes and patient satisfaction. Centralized information, a communication form that stays with the patient, better links between facilities and providers, and practice guidelines were suggestions shared by AHPs and physicians as ways to address this issue. 'Team care' was another approach to enhance communication and was acknowledged as more feasible in inpatient rehabilitation settings where different healthcare providers were housed under the same roof, shared charting and participated in regular team meetings. Inadequate staffing, part time positions and staff turnover negatively impacted team dynamics and consistencies in care. A lack of a collaborative, multidisciplinary approach was felt to lead to inefficiencies, duplication of services and patient dissatisfaction.

*"The problem in our health care system is that the bureaucratic aspect of things precludes us from being efficient..." *[36 M, SURG]

Patients suggested that surgeons could improve their communication and understanding of what is important to patients by:

*"Giving more time and listening to the patient. Assessing what they're saying, what the patient is saying. To give the patient time so that they feel comfortable enough to really express themselves." *[73 F, TKA]

A good patient-provider relationship and open communication were believed to motivate the patient and facilitate recovery. Suggestions for opening channels of communication included providing patients with contact phone numbers, calling them when they had missed appointments and liaising with the next health care provider in the rehabilitation continuum to ensure timely and efficient transfers of care.

*"One of the things I feel is really important is that physiotherapy departments and physicians don't forget their patients. ...call and see what's going on. Many people seem to feel like they were forgotten and that after physio and they were out on their own, nobody cared." *[73 M, TKA]

### Theme 2: Expecting the unexpected

Patients identified a number of unexpected challenges in the post-operative period for which they felt inadequately prepared: pain intensity and management, sleep disturbances, psychological issues and unrealistic activity expectations.

*"Nobody said how much pain and swelling there was going to be." *[76 F, TKA]

*"I think a lot of surgeons forget you've got to sleep - honest to God, they should have to go through it. The first thing is you'd be offered, you know, adequate pain medication post-operative and then that sleep is the biggest factor that you're faced with." *[73 M, TKA and retired health professional]

*"I don't know how many people [with TKA] I've had in the last little while that come in and they're stunned that they have pain postoperatively...They're so not prepared for the amount of pain they have." *[43 F, PT]

*"...after surgery I felt like the bull AND the china shop. Like I feel I am potentially the source of my demise and I feel fragile." *[57 F, THA]

Of equal concern to many patients and health professionals were the issues of who to go to when post-operative pain was not well-managed and inconsistent advice on whether additional analgesics (e.g. narcotics) were appropriate after the initial acute care period.

*"I don't think anybody tells the patients, so they go home, they'll be getting some T3's or something by their surgeon or surgical RN and sometimes that's enough, but usually it's not enough... and they just don't think to call or they don't know who to call." *[41 F, FP]

*"...the knowledge of pain management from the patient's perspective and their primary care provider's perspective is very poor." *[55 M, SURG]

All study participants viewed the pre-operative education and preparatory phase as being critical for clarifying expectations and empowering the patient.

*"What I've noticed is the [acute care] discharges tend to go better if patients are clear on the expectations, you know, that they're informed of the possible date of discharge so psychologically they can start to prepare themselves. Involving social work early on to assist with addressing the barriers or obstacles I find goes well* [42 F, SW]

Unclear or unrealistic patient expectations were felt to lead to greater post-operative pain, significant anxiety and depression, and disappointment around the rate of recovery.

*"I think my expectations on the recovery period were overly optimistic." *[57 M, TKA]

*"They should be realistic in what they project for you." *[69 M, THA]

Differing expectations and views between surgeons and rehabilitation providers on patients' functional status, ongoing need for supervised physical therapy and achievable outcomes lead to inconsistent advice, patient confusion, premature discontinuation of therapy and less than optimal outcomes. A PT described a common scenario whereby the surgeon's assessment differed from that of the treating therapist.

*" [The surgeon tells the patient at the 6-8 week follow-up visit] 'Oh, you're doing great. You don't need to do anymore (physical therapy).' Well, they're not doing great. I don't think they're gotten the best bang for their buck as far as the surgery, and ... you'd like to see them progress a lot further than they are..." *[43 F, PT]

Health professionals voiced concerns about misinformation available through the popular press and commercial Internet sites and said this was a growing problem leading to unrealistic expectations and a negative impact on patient recovery.

*"Patients learn just enough to be dangerous [from the Internet]." *[39 M, PT]

### Theme 3: It's attitude that counts

Health providers and patients alike stressed the importance of the patient's attitude when it came to being an active participant in the rehabilitation process and remaining motivated during the typical ups and downs of recovering from TJA surgery. Physicians and AHPs felt a key part of their roles was to help the patient in this regard: *"I like to empower the patient first and foremost." *[47 M, PHYS]

Patients were considered an integral part of the team and their active participation in the rehabilitation process vital to good outcomes and greater satisfaction.

*"I tell them 'This is what you need to do at home' and they go home and don't practice, definitely that makes a huge difference when you see the patient next time. People are afraid to move or people are really reluctant to do it, so I think patient compliance with home exercises is very effective, it's huge." *[42 M, PT]

*"I would think that a person should be checked to make sure that they are continuing to exercise, they are using the leg. I think it's such a waste of money and time if you don't become better." *[61 F, TKA]

Having a positive attitude and taking a proactive approach to the surgery and subsequent rehabilitation phase while acknowledging the mind-body connection were strategies used by many patient participants.

*"I learned to recognize that my body was wiser and far cleverer than I was so I had better just obey it." *[77 M, THA]

*"You have to be willing to give not just 100 percent but 150 percent to your own recovery." *[46 F, THA]

### Theme 4: It takes all kinds of support

Participants reported how different 'facets' of support contributed to health outcomes and overall satisfaction with the surgery and rehabilitation process. Patients and AHPs were more likely than physicians to describe peer and spousal/family support as having favorable effects on an individual's rehabilitation process.

*"Hearing from another patient first hand and how they experienced it really helps the fear part of it." *[43 F, RN]

*"The support from my spouse and my family immediately after surgery was the most invaluable and wonderful. Because we are sent out of the hospital faster now and you've just got to have that help at home." *[64 F, TKA]

The important role of family was acknowledged in descriptions of one health care facility where a family member was given the designation of 'coach' and encouraged to participate in all aspects of the patient's rehabilitation. Involving a family member was also ideal in cases where cultural differences and language barriers impeded rehabilitation instruction. When spousal and/or family support was lacking, there was greater need for home support services. In several communities, a lack of such services coupled with few transitional care units/beds was felt to contribute to longer acute hospital stays and a group of patients "who fall into the cracks".

Patients wanted to be recognized as a whole person and valued a holistic approach, which was sometimes lacking. Patients shared stories of how feeling supported enhanced their recovery and coping.

*"After the [physical therapy] program she phoned me and asked me how I was doing, so that was pretty good. It gives a little bit of feedback to the people and they feel inside that at least somebody cares about them." *[58 M, THA]

Physicians discussed their role in supporting and counseling TJA patients, however, both family practitioners and specialists expressed concerns over their ability to spend sufficient time with patients. The 'system' was most often blamed for not allowing for protracted conversations with patients: *"Physicians don't get paid adequately to provide counseling on an ongoing basis to patients." *[62 M, FP] Patients also expressed their frustration in accessing their surgeon post-operatively.

*"Does anyone find it important to have access to your surgeon, which is almost impossible? Anything, just hearing him, you know, on the phone even. Maybe you want to say something that's been bothering you and I'm sure you're not the only one that's ever bothered, but you feel reassured." *[75 M, TKA]

Another area of professional support overlapped with communication concerns; it was believed that health professional advice and guidance should be more consistent to be helpful:

*"... and I know that we can't all give the same exercises but I think everyone - we all have slightly different messages, we say slightly different things as to how long it's going to take or talking about the wound or talking about pain management. It would be really good if we could have some sort of education or something that's a little bit more consistent as far as the message that's going out for people." *[43 F, PT]

*"They're not standardized. I'm just thinking, there's all sorts of physiotherapy clinics around and they all do different kinds of things...." *[57 F, THA]

### Theme 5: Barriers to recovery

Participants identified patient, provider and system level factors as being barriers to recovery after TJA. Patient factors such as pain coping, motivation, attitude, state of readiness for treatment, psychological distress and self-efficacy were felt to influence the acute care hospital stay, course of recovery and participation in rehabilitation.

*"...pain management after total knee replacement is probably one of the biggest barriers to recovery." *[55 M, SURG]

*"One of the most common [concurrent] diagnoses that gets noticed is depression in the patients ... which hugely affects motivation, adherence to the protocols, and follow up, and it doesn't get addressed frequently because primary care physicians don't take the time to diagnose it appropriately. It's probably the most widely under diagnosed and under treated condition." *[53 F, RN]

Physicians and surgeons saw the role of rehabilitation after TJA as being *"to enhance the safety of the [surgical] procedure and make it easier for the patient to recover." *[64 M, SURG] However, the quality of rehabilitation, and in particular physical therapy services, was frequently thought to be poorly administered and therefore more detrimental to patients' recovery than helpful.

*"I have little faith in the ability of the external providers to provide appropriate care for my patients and I tend to dissuade them from pursuing outpatient physical and occupational therapy after surgery. ...my experience has been that they [therapists] tend to do more harm than good." *[55 M, SURG]

While several surgeons described having a good relationship with rehabilitation professionals and expressed confidence in their referral to post-operative physical therapy services, others did not: *"We are sending them into a dark, black hole." *[60 M, SURG]

At both patient and provider levels, language barriers and lack of translated educational materials were believed to compromise AHPs' ability to provide effective and timely education and support in a variety of rehabilitation settings. At the system level, issues related to access to rehabilitation were common to both Canadian and American participants; however, the contributing factors differed in important ways. Prolonged waits for surgical consultation, TJA surgery and in some cases, outpatient rehabilitation were unique to Canadian experiences.

*"...the Canadian system should be very clearly differentiated from the American. Their healthcare system is totally different. There's no similarity at all. ...we have the longest waiting list in the Western world." *[60 M, SURG]

*"...when it comes to the physio after, there don't seem to be more physiotherapy spaces. We all experienced longer waits. And we've all felt we've developed slower because of this extra wait." *[64 F, TKA]

Caps on physical therapy and rehabilitation services through private health insurers and managed health care practices were at issue in the American experience. Limited healthcare resources, ever-changing funding formulas and costs of rehabilitation services concerned all stakeholder groups in both countries.

*"If [patients] don't do physio it's usually because it's going to be expensive and they don't have extended health [insurance]." *[41 F, FP]

*"Medicare has put a cap on the amount of money that you can get in terms of the physical therapy and I think that's wrong. People vary too much in how they respond to surgery and to put a dollar value on that is totally crazy." *[72 F, TKA]

With limits on access to supervised rehabilitation, patients and providers had to decide how and when to use their 'allotment'. While some surgeons routinely sent people for physical therapy before surgery (pre-hab), others felt that rehabilitation postoperatively was of greater value. The duration of rehabilitation follow-up care was also curtailed by such funding caps.

Barriers to rehabilitation services included limited access outside urban settings and larger hospitals. Patients typically had fewer if any options for publicly funded therapy in more remote areas of Canada. Travel and associated costs with receiving rehabilitation outside of their home community were problematic for patients.

*"It's been hard because I live so far away. It's about a two and half hour drive from here to [my rehabilitation setting]." *[51 M, TKA]

*"I think the farther you get away from a hospital and whether you're talking doctors or physiotherapists, oftentimes you do move away from evidenced based practices..." *[63 M, PHYS]

Suggestions for addressing issues related to access and quality of care in rural communities included greater use of tele-rehab and enhanced training for rehabilitation providers.

### Theme 6: Back to normal

This final theme reflects the common view that patients wanted nothing more than to return to a sense of normalcy after surgery. While being pain-free and mobile was of primary importance, a more holistic view of 'normal' was repeatedly expressed.

*"I want to get back to be able to walk distances and participate in cross-country skiing, snow shoeing and hiking and fitness class, you know, things I did before." *[76 F, THA]

*"...I can only think of emerging from this cocoon of pain, which pulls you into a very small horizon. And so I really just wanted to get my vitality back." *[77 M, THA]

*"...to do my work is really just life's blood to me." *[64 F, TKA]

*"I was on crutches for 4 years and I have an 8-year-old daughter, so she'd never really seen me walk without crutches and now I don't have them. So that was really important. She sees me more as a normal person - now I can be the parent again." *[46 F, TKA]

*"...you don't want people losing their independent community skills so that they can stay out of nursing homes." *[53 F, RN]

There was strong support for a holistic approach to conceptualizing and measuring outcomes from the patients' perspective.

*"Look at the whole person. The psychosocial aspect is not always surgeons' strong suit." *[63 F, TKA]

*"...I've had times where I felt that everyone had an area of expertise and that me as a whole person, nobody was addressing or even wanting to hear about the total person going through this." *[64 F, TKA]

Consistent with the diverse conceptualization of 'normal' as the desired outcome, ways of measuring outcomes varied greatly with no agreement on measurement approaches or the value of using standardized tools in clinical settings. Measures that could be used throughout the rehabilitation continuum were thought to be ideal.

*"...it would be nice for people to actually use the same outcome measures pre-operatively, immediately post-op... so you could actually see a difference." *[43 F, PT]

*"Some people I believe use the WOMAC. Some people use the Oxford. Some people have their own little compilation of different things, and I really don't know what they use off in private practice frankly. So big weaknesses and we don't have a standardized approach to this yet." *[63 M, PHYS]

Others questioned the value of administering outcome tools and questionnaires.

"*...I think that you have got to be very careful about trying to quantify it at all. Questionnaires, I've come across them before and I think this is stupid! And you put something down, you don't know how it's going to be interpreted." *[81 M, TKA]

"*I don't ask patients to fill out questionnaires. That's highly inefficient." *[56 M, SURG]

When prompted to discuss the need for ongoing follow-up or long term monitoring of patients' outcomes, with the exception of surgeons, most felt that surgeons, primary care physicians and AHPs should be involved in follow-up care. Physical therapists were named most often as being able to offer an important complementary role to the surgeon's evaluation.

*"The same team should follow the same patient, because the [surgeon] now, what's the first thing he does? "Okay, your x-ray looks great." But the patient says, "I'm not walking good." We don't treat x-rays, we treat people, right?" *[42 M, PT]

Across all themes were the views that not only were there different patterns of recovery, rehabilitation and outcomes following THA and TKA surgery, but also a need to consider individual variations. Younger or more active individuals have different outcome expectations and rehabilitation needs than older or more sedentary patients.

## Discussion

This paper describes the results of the initial exploratory phase of a mixed method project to develop practice guidelines for THA and TKA rehabilitation. A pragmatic approach was used to identify recurrent issues and important concepts for each of the broad discussion points in order to inform guideline development and ensure stakeholders' views were captured at the outset. A lack of communication coupled with poor appreciation for each other's roles and expertise appeared to be major issues among our study participants. This was most apparent with family physician-surgeon and PT-surgeon dyads, in less rural communities and between health care settings. Trust was also a dominant factor with many surgeons sharing concerns about the quality and safety of treatment approaches thought to be provided by outpatient PTs; PTs also lacked trust about other PT providers. Lack of trust could potentially be alleviated by improved communication to reduce the misunderstandings, conflicts, inefficiencies and role confusion that may arise and severely hamper patient care and outcomes [15,32-34]. Different professional training and cultures may explain some of the disparity in how health professionals communicate.

For change to occur, it will require support at both the provider and system level [35]. Greater opportunity for inter-professional dialogue is needed to truly enact team care within programs and across the continuum of care. Patients' perception of poor and inconsistent communication among their healthcare providers can negatively impact patient adherence, confidence, outcomes and satisfaction [36]. Participants in this study spoke to the need for patient-provider communication to improve professionals' understanding of patients' beliefs and preferences and clear, shared expectations regarding rehabilitation outcomes of TJA surgery. Disparities in expectations and evaluation of surgical outcomes are well documented with surgeons tending to rate outcomes more favorably than patients [13,14]. The intensity and duration of post-operative pain was common yet unexpected among patient participants in our study. Despite this information being readily available through previous studies [37-41], inadequate provider-patient communication and education may once again be at fault. We found there was both uncertainty and disagreement among patient and physician participants concerning professional responsibility for ensuring adequate pain control beyond the immediate post-operative period. Similarly, sleep disturbances described by patients in our study have been previously reported [39,42] yet not adequately covered in pre-operative education sessions and virtually ignored during the early recovery phase. This was problematic for both patients and their spouses.

Post-operative anxiety, depression, fear and vulnerability were widely reported by patients and of concern to many AHPs. While pre-operative psychological factors were not specifically probed in our study, the literature suggests that pre-operative depressive symptoms are strongly related to post-operative outcomes and satisfaction [37,38,41,43]. Study participants recommended pre-operative screening for depression and other factors that may contribute to protracted pain and psychological distress and improved surgeon awareness of such psychological factors.

Emotional well being including more positive attributes (e.g., self-worth, hope, confidence, empowerment) is increasingly recognized as an important factor in coping and health outcomes of a number of chronic conditions and surgical procedures [36]. Few studies examining the role of patient factors' in determining TJA need and outcomes have included these attitudinal factors in their analyses of important characteristics. Further, current orthopaedic outcome tools fail to capture the concepts of patient attitude, self-efficacy and empowerment [38] despite the evidence suggesting self-efficacy, for example, impacts patient expectations [44], long-term functional outcome [45] and adherence to prescribed exercise [46]. Poor adherence was a commonly held assumption of AHPs in our study and felt to be strongly related to patients' overall attitude about their role and outcome expectations of rehabilitation. Adherence to therapeutic protocols is problematic in many studies of TJA rehabilitation and warrants subgroup analysis to determine whether higher adherence (e.g., greater treatment dosage) results in larger treatment effects. These findings support adoption of self-efficacy theory to guide interventions, such as adopting efficacy enhancing strategies like contracting and role-modeling to enhance patient's confidence regarding the adoption of habits that will support their recovery [47].

Our findings show that undergoing TJA surgery magnifies the need for support in the short term, consistent with other qualitative reports regarding the value of family and peer support to patients post-operatively [23]. Better social support is associated with lower complication rates, better functional outcomes and higher post-operative quality of life [48]. Patients described feeling supported by health professionals when they were 'heard' and given sufficient time to have their questions and concerns addressed. Similarly, health professionals were most satisfied with their support efforts when they had adequate time to spend with the patient. Surgeons on the whole admitted to having little time to provide the support and guidance sought by most patients and this is equally problematic in Canada and the US.

Concerns about poor health professional support were linked mostly to the follow-up (FU) phase, once supervised rehabilitation was completed. While the patients in our study had a TJA within the past year, several had undergone TJA surgery on another joint previously and expressed their dissatisfaction and feelings of being forgotten after rehabilitation ended. In a survey and chart review of 622 THA patients from three US states, only 41% reported consistent FU visits with their orthopaedic surgeons over a 6-year period and 16% reported they had no FU care [49]. Older individuals and those with lower socioeconomic status were less likely to receive regular FU. Our study patients suggested they would feel more supported in the year following TJA with regular phone calls, drop-in FU clinics with both surgeons and PTs, and group classes to review exercises, monitor progress and address any concerns.

Personal, provider and system-level factors were identified by our study participants as creating barriers to patients' recovery after TJA. Hoppe *et al*. acknowledged rehabilitation as an important tool in reducing costs of disability regardless of cause [50]. However, with "the rapid proliferation of private rehabilitation services currently operating with little regulation" [pg 18], those using, prescribing and paying for the services are finding it increasingly difficult to determine if in fact, these services are of good quality, justified and cost-effective [50]. In addition to other strategies, routine use of outcome measures and practice guidelines is suggested as a means of justifying and standardizing treatment approaches to address the structure, process and outcomes of the rehabilitation system. Capping the number of visits or duration of rehabilitation may help to control costs but as identified in our study, such limits were felt to hinder the rehabilitation process, ignore individual patient needs, and potentially lead to poorer outcomes and an overall increase in direct and indirect costs [50].

The issue of timely access to surgical care has been a priority of provincial healthcare ministries in Canada for several years and the focus of several innovative quality improvement strategies [34,51,52]. However, little attention and additional funding have been directed toward addressing barriers to quality rehabilitative care following surgery. Access, including transportation concerns, to rehabilitation services continues to be problematic for Canadians and Americans living in more rural settings. Greater use of technology including telerehabilitation (e.g., videoconferencing, remote monitoring) was voiced as a possible solution and deserves further investigation in this patient population [53].

Sanderson *et al. *reported clinicians and patients have different perspectives on outcomes and whereas patients' conceptualization of valued outcomes is broad, health professionals tend to focus on pathology and functional disability [54]. We found a similar trend with patients describing a wide range of anticipated and expected outcomes covering many dimensions of health and psychosocial well-being while health professionals, in particular physicians and surgeons, focused more on impairment, basic function (e.g. walking, using stairs) and surgical parameters (e.g., fixation of implant). These incongruent views may play a role in the reported discrepancies between patients' and health professionals' evaluation of surgical outcomes in which there are moderate correlations at best between patient and clinician assessment of symptoms and disability [55].

Few health professionals reported routinely using standardized outcome measures in their surgical and clinical practices, despite considerable support for their use. Participants' negative views on the utility (e.g., meaningfulness of numerical scores) and feasibility of using such instruments in clinical practice (e.g., time to administer and score) contributed to the low rate of standardized outcome evaluation. Jette *et al. *reported that a lack of support (e.g., technology, staffing) and irrelevant and confusing questions were barriers to routine use [56]. Further, the apparent confusion among health professionals regarding what constituted an outcome measure may have led to underreporting and suggests more education is needed.

Racial differences in patient-provider communication and the expectations and utilization of joint replacement therapy have been described elsewhere [57,58], however, we could find no published data specific to the experiences of Aboriginal North Americans undergoing TJA. The isolation and lack of access to TJA rehabilitation care described by the one First Nations person living on reserve in our study may reflect geographical, racial or other differences and warrants systematic study, in collaboration with aboriginal communities.

With the overarching views that "hips and knees are two different beasts" and subgroups of patients require different rehabilitation approaches, it is important to avoid a 'one size fits all' approach when designing rehabilitation practice guidelines for a broad target audience.

### Strengths of the study

The credibility and trustworthiness of findings were enhanced by using a standardized discussion guide, multiple data sources, peer and member checking, independent coding and maintenance of an audit trail throughout the data collection and analyses phases. This study provides new data on specific inter-professional communication issues and barriers to recovery after TJA and shares insight from two vastly different health care systems. Further, it adds to the research on protracted post-operative pain, sleep disturbance and anxiety well beyond the immediate post-operative stage, which all stakeholders agree are inadequately and inconsistently managed. The perspectives of patients and health care providers alike are important to ensuring the relevance of practice guidelines, which are extremely time-consuming and expensive to produce [59] and it is imperative to guideline adoption that all viewpoints be carefully considered.

### Limitations

Due to delays in the ethical review process incompatible with project timelines, only one US site was involved. It is unlikely that we heard the diversity of experiences and health care delivery issues that are inherent in a country with no universal healthcare program and varied access to health insurance. As well, the attitudes, functional limitations, access to specialty care, and rehabilitation experiences of uninsured individuals were not captured and may differ from the individuals in our study. Secondly, physician/surgeon focus groups were challenging to organize and did not include as much practice setting diversity as intended. Physicians' views may not be transferable to those practicing in more rural settings with less access to rehabilitation resources for their patients. Similarly, despite efforts to ensure maximum diversity in patient participants, the experiences of less educated individuals and those not receiving formal rehabilitation services were underrepresented.

### Clinical implications

There are several take home messages for clinicians, most of which are directly aligned with principles of client-centered practice [60] aiming to individualize intervention for optimal client outcomes as well as best use of therapeutic resources:

• Prior to surgery, ensure patient and provider expectations are clearly communicated and realistic;

• Prior to surgery, develop a plan for addressing post-acute pain management, psychological distress and sleep disturbances for several weeks following surgery;

• Use strategies to enhance self-efficacy and empower patients to adopt a positive attitude and take an active role in their rehabilitation;

• Incorporate efficient approaches to optimize health professional support and follow-up care beyond three months after TJA;

• Where possible, engage family members and peers in education, counseling and exercise instruction;

• Select meaningful outcome measures and consistently use to evaluate effect of interventions throughout the care continuum and across health care settings.

### Future research directions

This study raises a number of questions that could be addressed through future research including an examination of communication and information technologies (e.g., telerehabilitation) on patient-provider and inter-provider communication and delivery of TJA rehabilitation services. Development and testing of a decision aide or screening tool would assist health care providers in identifying patients at risk for protracted pain, emotional distress and functional impairment. Further, there is a need to design, implement and evaluate the effects of a range of FU programs on patient satisfaction and long-term outcomes after TJA.

## Conclusions

This qualitative, exploratory study provides valuable insight into rehabilitation experiences, attitudes and expectations of individuals who have undergone THA or TKA surgery and the health professionals directly involved in their care. Patients offered a perspective that differed, but overlapped, with the perspectives of health professionals regarding rehabilitation practices and outcomes. Themes arising from all stakeholder groups related to communication, unexpected events, importance of patient attitude and active involvement, professional and social support, barriers to recovery and a return to normalcy. Awareness of the facilitators and barriers to achieving optimal outcomes that emerged from this study will help clinicians and administrators in the design and delivery of pre- and post-operative interventions aimed at helping patients reach their desired goals after TJA. Stakeholders' views on rehabilitation for TJA will inform the next phases of guideline development and ensure all perspectives shape guideline priorities, scope, and format.

## Abbreviations

AHP: Allied Health Professional; FP: Family Practitioner; FU: Follow-up; KIN: Kinesiologist; OA: Osteoarthritis; OT: Occupational Therapist; PT: Physical Therapist or Physiotherapist; PHYS: Physiatrist; RHEUM: Rheumatologist; RN: Nurse; SURG: Surgeon; SW: Social Worker; THA: Total hip arthroplasty; TJA: Total joint arthroplasty; TKA: Total knee arthroplasty; US: United States

## Competing interests

The authors declare that they have no competing interests.

## Authors' contributions

MDW and CLB conceived and designed the study. MDW conducted a majority of the focus groups and interviews and both authors analyzed the data. MDW drafted the manuscript and both authors read, revised and approved the final manuscript.

## Appendix A - Discussion guide for health professionals

### Key questions

1a) Think about these services or programs you are involved in. What is working well?

Probes:

What allows (enables) you to provide good care to these clients?

What aspects of your rehabilitation care wouldn't you change?

1b) Still thinking about these rehabilitation services, tell us what isn't working well?

Probes:

What aspects of care would you change?

Are there any concerns that you have regarding rehabilitation services available to patients following these surgeries?

What gets in the way (barriers) of providing best care to these clients?

2) We are now going to shift from talking about rehabilitation issues and look more closely at outcomes after THA and TKA. What outcomes do you feel are important following THA and TKA?

Probes:

Think of both short-term and long-term outcomes, rehabilitation and surgical outcomes,

impairment, activity and participation levels

3) How should these outcomes be assessed or measured in the clinical setting?

Probes:

Do you use any self-report measures? Health professional scored tools? Performance measures?

4) Information from these focus groups will contribute to the larger project of developing multi-disciplinary clinical practice guidelines for THA and TKA rehabilitation. There are a lot of different ways that we could share the final results or recommendations with you. How would you like to get this information? [Results of this fourth discussion point will appear in a separate paper.]

Probes:

What would be most helpful to you?

In what format? (written, verbal, interactive, audiovisual)

In how much detail? (detailed report, summary, quick study guide)

## Pre-publication history

The pre-publication history for this paper can be accessed here:

http://www.biomedcentral.com/1472-6963/10/119/prepub
